# Regional differences in renal replacement therapy in northern Norway 2000–2012

**DOI:** 10.3402/ijch.v74.24298

**Published:** 2015-02-10

**Authors:** Jan Norum, Torbjørn Leivestad, Bjørn Odvar Eriksen, Siw Skår, Anne Fagerheim, Anna Varberg Reisæter

**Affiliations:** 1 Department of Clinical Medicine, Faculty of Health Sciences, UiT – The Arctic University of Norway, Tromsø, Norway; 2 Department of Radiology, University Hospital of North Norway (UNN), Tromsø, Norway; 3 Norwegian Renal Registry, Section of Nephrology, Department of Transplantation Medicine, Oslo University Hospital, Rikshospitalet, Oslo, Norway; 4 Section of Nephrology, University Hospital of North Norway, Tromsø, Norway; 5 Northern Norway Regional Health Authority Trust, Bodø, Norway; 6 Nordland Hospital, Bodø, Norway

**Keywords:** Norway, renal replacement therapy, subarctic, survival

## Abstract

**Objective:**

Distance from residence location to a centre for renal replacement therapy (RRT) may influence patients’ quality of life and prognosis. Northern Norway constitutes 45% of Norway's landmass, but has less than 10% of the population.

**Methods:**

In this study, we analysed all patients in northern Norway consecutively registered in the Norwegian Renal Registry during 2000–2012. A total of 634 patients (Nordland County 321 patients, Troms County 215 patients and Finnmark County 98 patients) were investigated.

**Results:**

There were more smokers (31% vs. 22%) and patients with diabetes (32% vs. 22%) in Finnmark, but the difference did not reach statistical significance. Patients undergoing RRT and living in Finnmark County had a significantly worse outcome (P=0.03). The median survivals after initiation of RRT were 3.8 years (Finnmark), 6.4 years (Troms) and 5.4 years (Nordland), respectively. The most common causes of death were cardiovascular disease (53%), infections (16%), withdrawal from therapy (15%) and malignancy (13%). In a Cox analysis, age (P<0.0001), diabetes (P=0.008) and smoking at any time (P<0.004) were individual factors correlated with inferior prognosis.

**Conclusion:**

Age, smoking and diabetes were prognostic factors. Residents of the northernmost county (Finnmark) experienced an inferior prognosis. Long distance from residence location to hospital may be another factor, but this could not be documented. Preventive strategies should be improved.

Distance from residence location to a centre for renal replacement therapy (RRT) may influence patients’ quality of life and prognosis (1–3). Northern Norway covers almost half of Norway's landmass, but has only 9.4% of the total population of 5.1 million inhabitants. The region has a subarctic and arctic climate that may cause several challenges, especially during wintertime with cold weather conditions, seasonable darkness and lot of snow. These geographical and demographical challenges must be compensated for to achieve a high-quality RRT health care within reasonable costs and acceptable logistic solutions. In the most northern county of Norway (Finnmark), the population is scattered within a landmass (48,616 km^2^) 12.9% greater than Denmark. In this region, satellite dialysis units manned with specialised nurses have been linked to the university clinic in Tromsø and ambulatory nephrologists and telemedicine services have been cornerstones in the health care service to RRT patients and their relatives (4).

Recently, nephrologists have expressed concerns about the survival of patients undergoing RRT and living distantly from the main dialysis centre (1–3). Long distance has been associated with increased risk of *Staphylococcus aureus* peritonitis among patients undergoing peritoneal dialysis (PD) (1). Samuel and co-workers documented an association between residence location and likelihood of transplantation among paediatric dialysis patients (2). Similarly, Axelrod and associates (3) observed that patients living farther from the transplant centre and having lower socioeconomic status had inferior kidney transplantation access and outcomes.

The aim of this investigation was to analyse the treatment outcome of RRT patients in northern Norway.

## Methods

The Norwegian Renal Registry (NRR) was formally constituted in 1994 in collaboration between the Norwegian Renal Association (NRA) and Oslo University Hospital, Rikshospitalet, with the latter as the formal owner (5). All patients initiating chronic RRT in Norway are included in the registry.

**Fig. 1:**
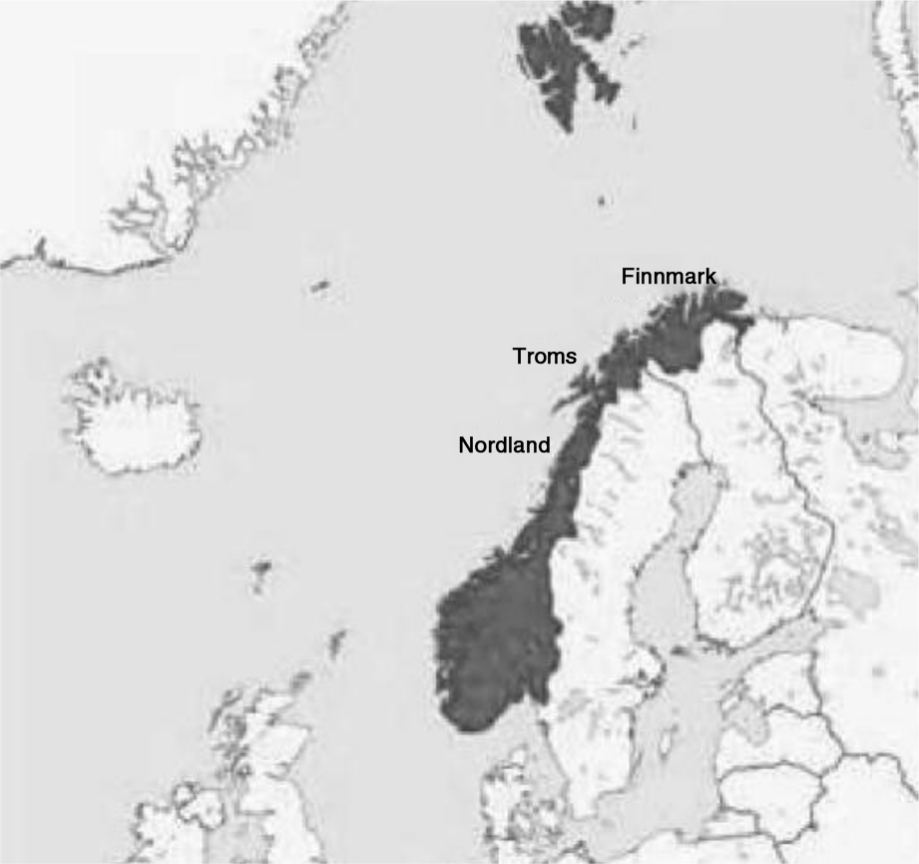
The figure shows the Scandinavian Peninsula with Norway in grey and the 3 northern counties of Norway marked with their names.

In this study, we focused on the survival of patients on RTT in northern Norway. This northern region consists of 3 counties (Nordland, Troms and Finnmark); the population in each county is respectively 240,000, 160,000 and 75,000 inhabitants. The location is visualized in Fig. 1. The region has 2 major centres for RRT located at the University Hospital of North Norway (UNN) in Tromsø and the Nordland Hospital situated in Bodø.

All patients included in the NRR and who had initiated RRT between 1 January 2000 and 31 December 2012 were included in the study. A total of 634 patients (Finnmark – 98 patients, Troms – 215 patients and Nordland – 321 patients) were identified. Details are shown in Table I. Treatment withdrawal was defined as patients’ and doctors’ decision of withdrawal from therapy.

**Table I:** Demographics, age, gender, smoking and diabetes among RTT patients in northern Norway according to county of residence at the time of initiation of therapy

Variables	Finnmark	Troms	Nordland
Patients (numbers)	98	215	321
Incidence rate (PMP/year)	103.6	107.4	104.6
Median age (years) [range]	65.7 (0.04–83.5)	64.8 (15.1–90.5)	67.7 (1.3–88.9)
Males (%)	75 (76.5)	150 (69.8)	223 (69.5)
Diabetes			
Diabetic nephropathy (%) total 85 patients	20 (20.4)	26 (12.1)	39 (12.1)
Diabetes total (%) total 150 patients	31 (31.6)	46 (21.4)	73 (22.7)
Smoking^a^			
Never smoked^a^ (%)	20 (26.0)	68 (37.4)	109 (39.8)
Active smoker^a^ (%)	24 (31.2)	39 (21.4)	61 (22.3)

None of the differences reached statistical difference. PMP=per million population.

a Smoking habits were only available for patients treated between 2005 and 2012. In total, 101 patients were excluded due to lack of data on smoking habits.

### Statistics and authorization

The comparison between the 3 counties of northern Norway was done using t-test and chi-square statistics. Survival was estimated employing the Kaplan Meier method and differences between counties in northern Norway were tested with the log-rank test. The time of observation was from start of RRT until death, recovery, emigration or censoring date 1 April 2013. The effects of residency on survival were explored employing the Cox regression analysis. Significance was set to 5% and the tests were carried out 2-sided. Statistical Package for Social Science (SPSS) version 16.0 was used for the database and statistical calculations.

All patients included in the NRR have provided written consent. In the present investigation, except for the colleagues employed at the NRR, co-workers had only access to anonymous and aggregated data. Consequently, no approval from the Regional Committees for Medical and Health Research Ethics (REK) was necessary.

## Results

There was no difference in mean age at initiation of RRT between counties in northern Norway. The incidence rate per million population (PMP) per year was 103.6 (Finnmark), 107.4 (Troms) and 104.6 (Nordland), respectively. Most patients were males (448 patients – 70.7%). The proportion of males, diabetics and smokers was the highest in Finnmark, but differences did not reach statistical significance. Details are shown in Table I. Only 21% of patients started with continuous ambulatory peritoneal dialysis (CAPD) or other forms of PD, and there were no differences between counties.

**Fig. 2:**
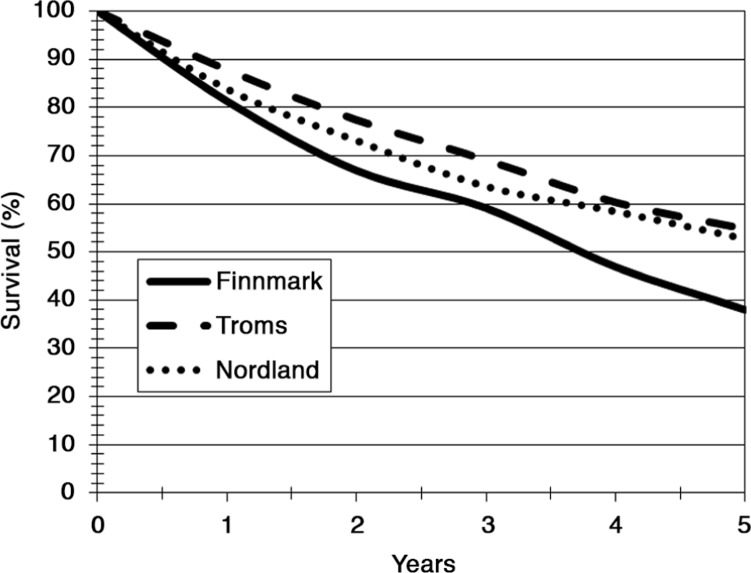
The figure shows the survival curves (Kaplan Meier method) of patients undergoing renal replacement therapy according to county of residence in northern Norway.

The median survivals after start of RRT were 3.8 years (Finnmark), 6.4 years (Troms) and 5.4 years (Nordland), respectively. Details are shown in Fig. 2. The most common causes of death were cardiovascular disease (CVD) (53%), infections (16%), withdrawal from therapy (15%) and malignancy (13%). Details are shown in Table II. A significant difference in overall survival was observed between Finnmark and Troms (P=0.006) and Finnmark and Nordland (P=0.04). There was no difference between Troms and Nordland (P=0.26). In gender-specific analyses (comparing Troms and Finnmark), the significant inferior survival rate in Finnmark was only observed among males (P=0.002).

**Table II:** The table shows mode of treatment, deaths and cause of death according to patients’ county of residence in northern Norway

Variables	Finnmark	Troms	Nordland
		
	No (%)	No (%)	No (%)
Patients	98 (100)	215 (100)	321 (100)
Started with PD	20 (20.4)	47 (21.9)	67 (20.9)
Predialytic transplantation	10 (10.2)	22 (10.2)	19 (5.9)
Achieved transplantation	35 (35.7)	100 (46.5)	124 (38.6)
Death (by May 2013)	55 (56)	91 (42)	156 (49)
Cause of death: number (% of deaths)			
Withdrawn from RRT	8 (15)	16 (17)	20 (13)
Cardiovascular disease	25 (45)	48 (53)	86 (55)
Infections	12 (22)	16 (18)	19 (12)
Malignancy	6 (11)	10 (11)	22 (14)
Other causes	4 (7)	1 (1)	9 (6)

PD=peritoneal dialysis; RRT=renal replacement therapy.

To further clarify the findings in Finnmark and Troms, we performed a Cox analysis. The analysis revealed age (P<0.0001), diabetes as primary disease (P=0.0082) and smoking at any time (P=0.0041) to be associated with inferior survival. The variable “active smoker” did not reach statistical significance. Smoking and diabetes combined outweighed county (Finnmark) as risk factor. Details are shown in Table III.

**Table III:** The table shows the covariates analyzed and Hazard ratio

Covariate	Hazard ratio	Hazard 95% CI	*p*
County (Finnmark vs. Troms)	1.596	0.922–2.208	0.1106
Age at start of RRT (by year)	1.104	1.07–1.129	<0.0001
Male sex	1.344	0.851–2.122	0.2045
Diabetes primary disease	2.221	1.230–4.011	0.0082
Smoking at any time	1.930	1.232–3.025	0.0041

Age, diabetes and smoking were associated with inferior patient survival. RRT=renal replacement therapy.

## Discussion

In this study, we have documented that patients undergoing RRT in our northernmost county (Finnmark) had a higher frequency of diabetes and smoking, and they experienced a significantly shorter life expectancy than patients from other counties of northern Norway.

### Strength and limitations

The strength of this study is that it included the total cohort of patients treated with RRT in northern Norway during the study period. Consequently, the prognosis of the total cohort is described. There are however limitations. The data on smoking at any time should be handled with some caution as there may be a potential bias related to missing information on smoking in 101 patients. It may also be discussed whether it is inappropriate to censor data in case of recovery. Data on uncensored mortality is discussed next.

### The survival figures

Differences in mortality in the general population may contribute to differences in mortality on RRT. It is well known that the people of Finnmark have the lowest life expectancy in Norway. The total mortality rate in the age group 0–74 years per 100,000 in Nordland, Troms and Finnmark was 291.1, 293.9 and 350.6, respectively, during 2002–2011 (www.helse-nord.no/helseatlasnord).

It could be claimed that a centre with high recovery rates will have an apparent poorer RRT survival simply due to shorter mortality-free exposure-time. In our study, the recovery figures of Troms, Finnmark and Nordland were 1% (2/213), 5.5% (5/93) and 5% (16/305), respectively. As we implemented data directly from the Norwegian Population Registry on all patients who have been included in the NRR, we could access data on uncensored mortality. Whereas the 1-year survival figures were 80.4% (Finnmark), 87.3% (Troms) and 83.1% (Nordland), the corresponding 5-year figures were 34.6, 50.2 and 47.7%, respectively. Comparing Finnmark and Troms counties, the difference was still statistically significant (P=0.0254). The difference between Finnmark and Nordland did not reach statistical significance (P=0.0622).

### Compared to other studies

Van Dijk et al. (6) revealed that only 26% of the European north–south mortality difference in RRT could be attributed to differences in general population mortality. It could be speculated that there is a north–south gradient within northern Norway. Such a gradient has been observed within Europe with a 35% lower mortality rate among patients on RRT in the south of Europe (6). The crude annual death rate of patients on RRT ranged from 200 per 1,000 patient years (py) in Northern Europe to 132 per 1,000 py in Southern Europe. The mortality on RRT was highest in Sweden (239 per 1,000 py) and lowest in Basque country (118 per 1,000 py). We have not revealed any explanation for this finding. However, when looking at our figures, it is only Finnmark County that deviates from the national figures. Troms County had, during the study period, the fifth best survival figure in Norway (personal communication Torbjørn Leivestad).

We revealed CVD, malignancy and infection as the most common causes of death among patients with end-stage kidney disease in northern Norway. This is in accordance with findings by other authors (7–9). Especially following kidney graft failure, infection has been reported the most common cause of death (7,8). Looking at our absolute figures, Nordland County had the lowest figure of deaths caused by infections (12%). Prior analyses have shown that the Nordland Hospital in general has low prevalence figures with regard to rate of hospital infections (10). In the future, retrospective analysis of electronic patient records (EPR) employing specific tools, for example the Global Trigger Tool (GTT), could be performed to clarify the kind and percentage of infections that can be avoided/prevented.

Among RRT patients, Finnmark County had the highest death rate due to fatal infections. Long distance has been associated with increased risk of fatal infections (1). We had no data on the bacteria or virus that caused fatal infections or the time from early symptoms to treatment. We are well aware of Finnmark by far being the largest county in Norway and its network of air ambulance resources available in the region. These resources may compensate for long distances and transport of patients and health care workers in emergency situations (10). The slightly lower risk of dying from cancer among RRT patients in Finnmark must be seen in the context of the short life expectancy observed among RRT patients. It takes time to develop cancer. The higher share of males in Finnmark may also have had an effect on the figures as males usually get cancer in older age than females do.

For decades, the association between tobacco smoking and lung and CVDs has been well known. Sánchez-Perales et al. (11) recorded acute myocardial infarction (AMI) among the patients treated in their dialysis unit in Spain between 1999 and 2007. The factors that predicted the occurrence of AMI in dialysis were older age, previous coronary artery disease and diabetes as a cause of nephropathy. Post-infarction mortality was very high and multi-vessel disease was very frequent.

The population of Finnmark lives in the northern subarctic area and frequently experiences the roughest weather conditions on the Norwegian mainland. They have 2 months with seasonable darkness and long-lasting winters. Cold weather increases the risk of CVD (12). Exposure to cold air causes a rise in blood pressure and haemoconcentration, which leads to increased tendency for vascular thrombosis (12,13). Added to the high percentage of smokers among RRT patients in Finnmark, it may look surprising that CVD was not a more common cause of death in this county.

The inferior prognosis among RRT patients in Finnmark was only observed among males. Different survival figures have been documented between genders (14–16). A few years ago, Eriksen and Ingebretsen (14) documented female gender associated with slower decline in GFR and better patient and renal survival. Furthermore, they concluded that the effect of gender on the progression of chronic kidney disease had been a matter of debate, where most of the evidence seemed to point towards a negative effect of male gender.

Studies have shown inferior survival among patients undergoing RRT and living distantly from the main dialysis centre (1–3). Samuel and co-workers documented an association between residence location and likelihood of transplantation among paediatric dialysis patients (2). Similarly, Axelrod and associates (3) observed patients living farther from the transplant centre and having lower socioeconomic status had inferior kidney transplantation access and outcomes. Compared to northern Norway and Norway in general, the people of Finnmark (both gender) have a shorter life expectancy (www.ssb.no). In our survey, the transplantation rate was highest in the university hospital region (Troms) and lowest in Finnmark. The care of patients undergoing RRT in Finnmark was administered and run by nephrologists at the UNN. The same practice with regard to transplantation and follow-up were employed in Finnmark as in Troms County. Differences in transplantation rate were due to different patient characteristics.

## Conclusion

In this study, we have documented that patients undergoing RRT in the most northern county (Finnmark) had the highest frequency of diabetes and smoking, and males experienced a significantly shorter life expectancy. Despite the fact that the higher frequency of diabetes and smoking did not reach statistical significance in Finnmark, these factors were documented individual prognostic factors. We therefore encourage smoking cessation strategies/campaigns and efforts to improve the treatment of diabetes. The latter may be improved by more frequent use of the NOKLUS Diabetes software (17) available in northern Norway's hospitals. This software provides decision support and data collection/overview and an almost complete diabetes medical record.
